# Exploring the role of MKK7 in excitotoxicity and cerebral ischemia: a novel pharmacological strategy against brain injury

**DOI:** 10.1038/cddis.2015.226

**Published:** 2015-08-13

**Authors:** A Vercelli, S Biggi, A Sclip, I E Repetto, S Cimini, F Falleroni, S Tomasi, R Monti, N Tonna, F Morelli, V Grande, M Stravalaci, E Biasini, O Marin, F Bianco, D di Marino, T Borsello

**Affiliations:** 1 Department of Neuroscience, NICO – Neuroscience Institute Cavalieri Ottolenghi, Neuroscience Institute of Turin (NIT), University of Turin, Turin I-10125, Italy; 2 National Institute of Neuroscience, Corso Raffaello 30, Turin, Italy; 3 Neuronal Death and Neuroprotection Laboratory, IRCCS – Istituto di Ricerche Farmacologiche, ‘Mario Negri', Via La Masa 19, Milan 20156 Italy; 4 Sanipedia, Via Ariosto 21, Bresso (MI) 20091, Italy; 5 Department of Biomedical Sciences, University of Padova, Padova 35121, Italy; 6 Fondazione Fernando Santarelli, Neuroinflammation Lab, Corso Venezia 18, Milan, Italy; 7 Department of Physics, Sapienza University of Rome, Rome, Italy; 8 Department of Chemistry, Brooklyn College, City University of New York, Brooklyn, New York, USA; 9 Department of Pharmacological and Biomolecular Sciences, University of Milan, Milan, Italy

## Abstract

Excitotoxicity following cerebral ischemia elicits a molecular cascade, which leads to neuronal death. c-Jun-N-terminal kinase (JNK) has a key role in excitotoxic cell death. We have previously shown that JNK inhibition by a specific cell-permeable peptide significantly reduces infarct size and neuronal death in an *in vivo* model of cerebral ischemia. However, systemic inhibition of JNK may have detrimental side effects, owing to blockade of its physiological function. Here we designed a new inhibitor peptide (growth arrest and DNA damage-inducible 45*β* (GADD45*β*-I)) targeting mitogen-activated protein kinase kinase 7 (MKK7), an upstream activator of JNK, which exclusively mediates JNK's pathological activation. GADD45*β*-I was engineered by optimizing the domain of the GADD45*β*, able to bind to MKK7, and by linking it to the TAT peptide sequence, to allow penetration of biological membranes. Our data clearly indicate that GADD45*β*-I significantly reduces neuronal death in excitotoxicity induced by either *N*-methyl-D-aspartate exposure or by oxygen–glucose deprivation *in vitro*. Moreover, GADD45*β*-I exerted neuroprotection *in vivo* in two models of ischemia, obtained by electrocoagulation and by thromboembolic occlusion of the middle cerebral artery (MCAo). Indeed, GADD45*β*-I reduced the infarct size when injected 30 min before the lesion in both models. The peptide was also effective when administrated 6 h after lesion, as demonstrated in the electrocoagulation model. The neuroprotective effect of GADD45*β*-I is long lasting; in fact, 1 week after MCAo the infarct volume was still reduced by 49%. Targeting MKK7 could represent a new therapeutic strategy for the treatment of ischemia and other pathologies involving MKK7/JNK activation. Moreover, this new inhibitor can be useful to further dissect the physiological and pathological role of the JNK pathway in the brain.

In many disorders of the nervous system, overactivation of *N*-methyl-D-aspartate (NMDA) receptors leads to neuronal death and consequent neurological impairment. NMDA-induced neuronal death, that is, excitotoxicity, has been implicated in many neurodegenerative diseases such as stroke, epilepsy, Alzheimer disease, spinal cord injury, traumatic brain injury, hearing loss, Parkinson's and Huntington diseases.^1^ However, the molecular mechanisms underlying excitotoxic neuronal death remain only partially understood.

Excitotoxicity triggers complex signal transduction events that induce the neuronal death program. Among them, activation of the c-Jun N-terminal kinase (JNK) pathway has a key role.^2, 3, 4, 5^ There are only two direct upstream activators of JNK: mitogen-activated protein kinase kinase 4 and 7 (MKK4 and MKK7).^6, 7^ In some cell types, MKK4 activates JNK primarily in response to stress stimuli, whereas MKK7 signaling is triggered by release of inflammatory cytokines.^8, 9, 10^ In neurons, however, we showed that MKK7 is mainly responsible for JNK overactivation during excitotoxicity both *in vitro*^3^ and *in vivo* following middle cerebral artery occlusion (MCAo).^4^ Conversely, MKK4 controls JNK physiological role and its activation is not affected by excitotoxic stimuli.^3^

Inhibition of the JNK pathway by the specific JNK inhibitor peptide, D-JNKI1, has been proposed for the treatment of ischemia.^2^ D-JNKI1 induces powerful neuroprotection in *in vitro* models of excitoxicity^2, 11^ and leads to a 93% reduction in the infarct size in rodent models of ischemia.^2, 4, 12^ Despite the potent and long-lasting neuroprotective effect of D-JNKI1, total inhibition of JNK is not deprived of negative side effects, as it regulates a variety of physiological events^13^ such as cell proliferation, survival and differentiation.^13^ For these reasons, MKK7 may represent a more attractive target for clinical application, as it activates JNK specifically after toxic stimuli. Thus, by targeting MKK7 the physiological role of JNK, regulated by MKK4, will be preserved.

Here we designed a set of new cell-permeable inhibitor peptides able to block MKK7 activity and protect against excitotoxic death.

We took advantage of the growth arrest and DNA damage-inducible 45*β* (GADD45*β*) ability to bind MKK7.^9, 14, 15^ GADD45*β* is involved in the control of cell stress responses in cell cycle, DNA repair and oncogenesis.^9, 16^ GADD45*β* binds tightly to MKK7 and inhibits its enzymatic activity^15^ by interacting with its catalytic domain.^9^ More importantly, GADD45*β* inhibition is MKK7-specific and has no effect on MKK4, MKK3/6 and MEK1/2 activity.^9^ The minimal essential domain of interaction between MKK7 and GADD45*β* has already been defined (GADD45*β*_60–86_ and _69–86_ sequences).^15^ We here used *in silico* approaches to design an effector peptide, based on the domain of GADD45*β*, and optimize its affinity for MKK7. We then linked the effector peptide to a TAT-cargo in order to penetrate neuronal plasma membrane.^17^ The selected cell-permeable MKK7 inhibitor peptide (GADD45*β*-l) confers neuroprotection *in vitro* against NMDA and oxygen–glucose deprivation (OGD) toxicity, as well as *in vivo* in two models of MCAo with a clinically relevant post-ischemic temporal window (6 h) at both 24 h and 1 week after lesion. These data shed light on a new approach for the treatment of ischemia.

## Results

### Design and development of TAT-MKK7 inhibitor peptides: GADD45*β*-I

**Figure 1:**
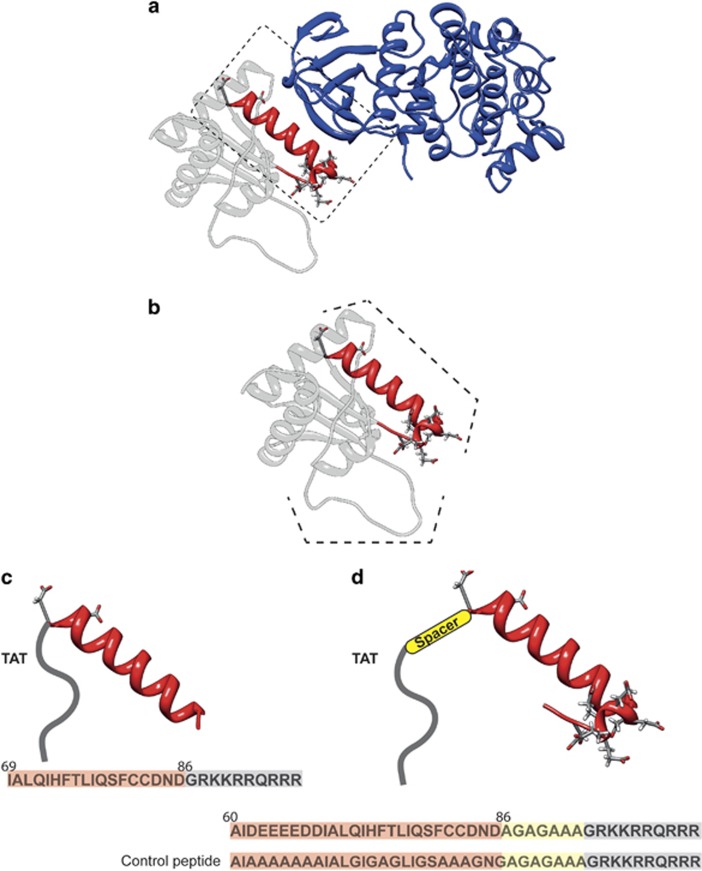
Design of specific inhibitors of MKK7 based on GADD45*β* structure. (**a**) Ribbon representation of the best complex resulting from the docking between the modeled structure of GADD45*β* and MKK7. MKK7 is shown in blue and GADD45*β* in gray. The helix-turn motif of GADD45*β* involved in the interaction, taken as a template to design the peptides, has been reported in red. (**b**) Structure of GADD45*β* protein obtained by homology modeling. Residues 60–86 forming a helix-turn motif are highlighted in red and with a dashed line. Residues 104–113 form a long loop with an alternation of hydrophilic and negative residues, this region is highlighted with a dashed line. (**c**) Modeling of TAT-GADD45*β*_69–86_ peptide. In red is the sequence of the *α*-helix (residues 69–86) and in gray the sequence of TAT. The entire sequence of the peptide is shown on the bottom. (**d**) Modeling of TAT-spacer-GADD45*β*_60–86_ peptide. In red is showed the sequence of the the *α*-helix (residues 60–86), in yellow the position of the linker and in gray the TAT sequence. The entire sequence of the peptide is shown on the bottom. The sequence of the TAT-GADD45*β*_69–86 CONTROL_ is also reported

The minimal essential region of GADD45*β* that interacts with MKK7 is at residues 60–86, but another region (residues 104–113) seems to have a more marginal role to stabilize the interaction between GADD45*β* and MKK7 (Papa *et al.*^15^) (Figure 1a). As shown in Figure 1a, reporting the structure of GADD45*β* obtained by homology modeling, residues 60–86 form a helix-turn motif (Figure 1b). Most of the residues in this region are hydrophilic and residues 62–68 are all negatively charged. Residues 104–113 form a long loop with an alternation of hydrophilic and negative residues (Figure 1b), and its marginal role in the GADD45*β*–MKK7 protein–protein interaction is probably due to the highly flexible nature of this portion of GADD45*β*.

GADD45*β* interacts with MKK7 in proximity of its ATP-binding site^9^ and this may justify the presence of acidic residues able to establish electrostatic interactions with the basic residues in this site.^9^

The docking results put in evidence that in the majority of the complexes obtained, region 60–86 is able to interact with MKK7 (Figure 1a), matching the experimental data already available.^9, 14, 15^ In detail, the *α*-helix of GADD45*β* establishes a network of hydrogen bonds with the *β*-sheets forming the MKK7 ATP-binding site (Figure 1a). The negatively charged residues following GADD45*β α*-helix interact through a cluster of electrostatic interactions with the positively charged residues of MKK7 (Figure 1a).

These pieces of information were used to design two peptides able to interact with MKK7 and inhibiting its function. The first peptide (GADD45*β*_69–86_) was designed by considering only the sequence of the *α*-helix (residues 69–86) and avoiding the cluster of negative residues (Figure 1c).

**Figure 2:**
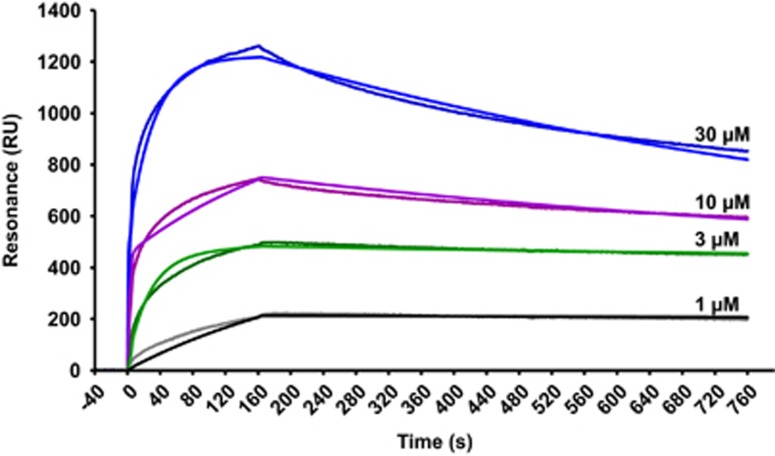
GADD45*β*_69–86_ interacts with recombinant MKK7. Starting at time 0, the indicated concentrations of GADD45*β*_69–86_ were injected for 150 s over SPR sensor surfaces on which 5000 RUs of human recombinant MKK7 had been previously captured by amine coupling. The SPR chip was then washed with buffer alone to monitor ligand dissociation. Fitted sensorgrams show GADD45*β*_69–86_ binding to recombinant MKK7 in RUs. The data were obtained by subtracting the reference channels, containing bovine serum albumin or no polypeptides. The heterogenous ligand equation was the best algorithm to fit the data, suggesting that recombinant MKK7 was immobilized in different orientations on the chip surface. Binding parameters obtained in this manner were as follows: *k*_on_=3.15 × 10^2^ 1/Ms; *k*_off_=1.30 × 10^−3^ 1/s; *K*_D_=3.71 *μ*M

Consistently with *in silico* prediction, GADD45*β*_69–86_ showed an affinity in the low micromolar range for recombinant MKK7 *in vitro*, as assayed by surface plasmon resonance (SPR, Figure 2). Of note, the measured binding affinity (3.71 *μ*M) of the GADD45*β*_69–86_ peptide for recombinant MKK7 was in good agreement with the biologically relevant concentrations observed in subsequent experiments. The TAT sequence was then been added to the N-terminal region of the peptide (Figure 1c). A second peptide (TAT-GADD45*β*_60–86_) was designed by considering all the regions formed by residues 60–86 (Figure 1d). As previously stated, residues 62–68 are all negatively charged and they could have a key role in stabilizing the interaction. However, this peptide showed poor solubility in water. To avoid the interaction between the negatively charged residues in the effector and the positively charged amino acids of the TAT peptide, a spacer sequence was added between the peptide and the TAT (TAT-spacer-GADD45*β*_60–86_) (Figure 1d). To validate the specificity of TAT-GADD45*β*_69–86_ and TAT-spacer-GADD45*β*_60–86_ peptides we designed a control peptide, TAT-GADD45*β*_60–86 CONTROL_, where 17 residues out of 27 composing the GADD45*β*_60–86_ peptide were changed. In detail, the control peptide was obtained by substituting all the negative charges with alanine residues (Figure 1d). Moreover, to achieve a complete inhibition of interaction with MKK7, we made the peptide less specific as possible by also mutating in alanine or glycine the amino acids with physical–chemical characteristics that could have a cooperative effect on the binding (Figure 1c; i.e., Gln, His, Phe, Thr and Cys, and Figure 1d). Based on these predictions, the control peptide TAT-GADD45*β*_60–86 CONTROL_ was designed in order to be an inactive peptide.

### Effect of GADD45*β*−I peptides on cultured neurons

**Figure 3:**
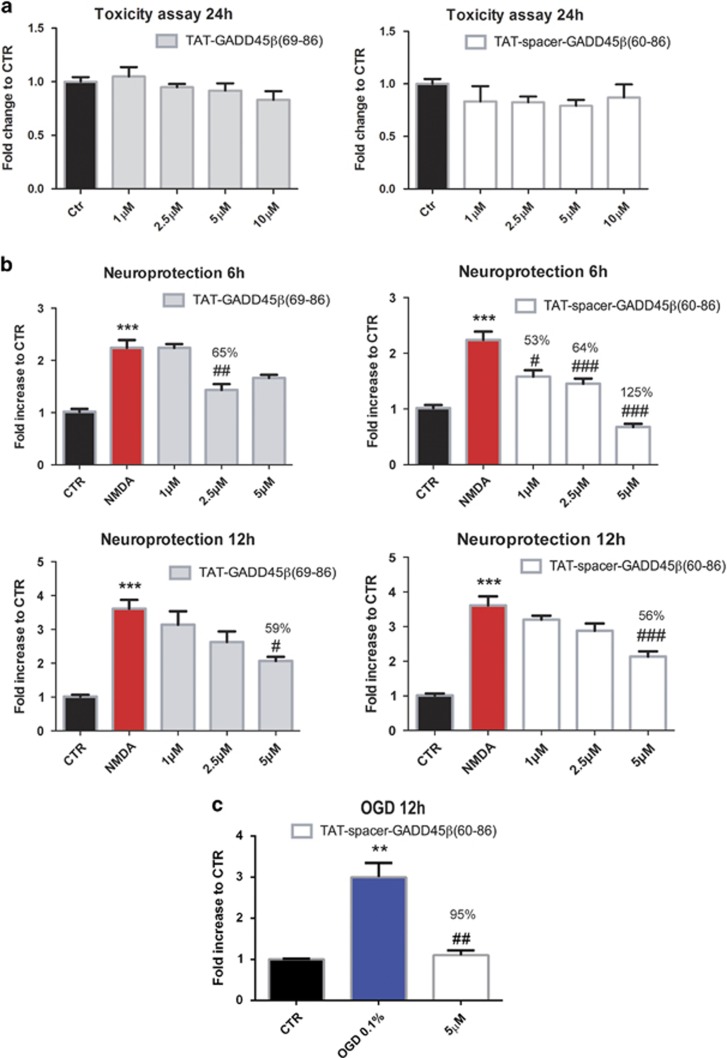
TAT-GADD45*β*_69–86_ and TAT-spacer-GADD45*β*_60–86_ do not induce neuronal death and protect neurons from NMDA and OGD insults. (**a**) LDH assay to assess intrinsic toxicity of MKK7 inhibitor peptides. Twelve DIV cortical neurons were treated with increased concentrations (1, 2.5, 5 and 10 *μ*M) of TAT-GADD45*β*_69–86_ (left) and TAT-spacer-GADD45*β*_60–86_ (right) for 24 h. Data are presented as mean±S.E.M. (one-way ANOVA, *P*>0.05, *n*=8). (**b**) LDH assay was performed on 12 DIV cortical neurons to evaluate the ability of TAT-GADD45*β*_69–86_ (left) and TAT-spacer-GADD45*β*_60–86_ (right) peptides (1, 2.5 and 5 *μ*M) to protect against 100 *μ*M NMDA-induced excitotoxicity *in vitro* for 6 (upper panels) and 12 h (lower panels). Data are presented as mean±S.E.M. (one-way ANOVA, Tukey's *post-hoc* test, ***P<0.001 NMDA *versus* CTR, ^#^*P*<0.05 NMDA+MKK7I *versus* NMDA, ^##^*P*<0.01 NMDA+MKK7I *versus* NMDA, ^###^*P*<0.001 NMDA+MKK7I *versus* NMDA, *n*=6). (**c**) LDH assay to evaluate the ability of TAT-spacer-GADD45*β*_60–86_ peptide pre-treatment (5 *μ*M, 30 min before OGD) to protect against 12 h OGD. Data are presented as mean±S.E.M. (one-way ANOVA, Tukey's *post-hoc* test, **P<0.01 OGD *versus* CTR, ^##^*P*<0.01 OGD+MKK7I *versus* OGD, *n*=6)

The cell-permeable MKK7 inhibitor peptides were tested in primary cultures from dissociated cortical neurons. Toxicity of the peptides was assessed after 24 h with LDH assay. Application of both TAT-GADD45*β*_69–86_ and TAT-spacer-GADD45*β*_60–86_ peptides did not induce neuronal death for concentrations as high as 10 *μ*M (one-way ANOVA, *P*>0.05) (Figure 3a). Conversely, the control (5′-AIAAAAAAAIALGIGAGLIGSAAGNGAGAGAAGRKKRRQRRR-3′) peptide, TAT-GADD45*β*_60–86 CONTROL_ application for 24 h resulted in an increase of neuronal death at the higher dosages (5 and 10 *μ*M) (not shown, one-way ANOVA, Tukey's *post-hoc* test, ***P<0.001).

### NMDA neurotoxicity and neuroprotection by GADD45*β*−I peptides

The TAT-GADD45*β*_69–86,_ the TAT-spacer-GADD45*β*_60–86_ and the TAT-GADD45*β*_60–86 CONTROL_ peptides were tested against NMDA toxicity *in vitro*. Addition of the cell-penetrating GADD45*β*−l peptides at the concentration of 1, 2.5 and 5 *μ*M protected neurons against excitotoxicity induced by exposure to 100 *μ*M NMDA for 6 h (Figure 3b). In fact, the TAT-GADD45*β*_69–86_ peptide exerts a 65% protection against NMDA insult at 2.5 *μ*M concentration, whereas the TAT-spacer-GADD45*β*_60–86_ peptide protects by 53, 64 and 125% at 1, 2.5 and 5 *μ*M, respectively (one-way ANOVA, Tukey's *post-hoc* test, ***P<0.001 NMDA *versus* CTR, #*P*<0.05 NMDA+MKK7I *versus* NMDA, ##*P*<0.01 NMDA+MKK7I *versus* NMDA and ###*P*<0.001 NMDA+MKK7I *versus* NMDA) (Figure 3b). Similar results were obtained after 12 h of NMDA exposure (Figure 3b). In this case, both TAT-GADD45*β*_69–86_ and TAT-spacer-GADD45*β*_60–86_ peptides lead to a significant protection of 59 and 56%, respectively, at the higher dose tested (5 *μ*M) (one-way ANOVA, Tukey's *post-hoc* test, ***P<0.001 NMDA *versus* CTR, #*P*<0.05 NMDA+MKK7I *versus* NMDA and ###*P*<0.001 NMDA+MKK7I *versus* NMDA). As expected TAT-GADD45*β*_60–86 CONTROL_ peptide showed no protection for all the concentrations tested (1, 2.5 and 5 *μ*M) and both time points (6 and 12 h) (Supplementary Figure S1, one-way ANOVA, *P*>0.05). Thus, the protective effect of the TAT-GADD45*β*_69–86_ peptides is entirely accountable to the peculiar amino acids composition of the region between residues 69–86 of the GADD45*β* protein.

Out of the two peptides tested, TAT-spacer-GADD45*β*_60–86_ resulted as the most efficient in preventing NMDA excitotoxicity. Therefore, we decided to use this peptide for the following experiments. We tested TAT-spacer-GADD45*β*_60–86_ in an *in vitro* model of OGD. TAT-spacer-GADD45*β*_60–86_ (5 *μ*M) inhibited by 95% the neuronal death induced by 12 h OGD (one-way ANOVA, Tukey's *post-hoc* test, **P<0.01 OGD *versus* CTR and ##*P*<0.01 OGD+MKK7I *versus* OGD) (Figure 3c).

### Efficacy and specificity of GADD45*β*−I peptides

**Figure 4:**
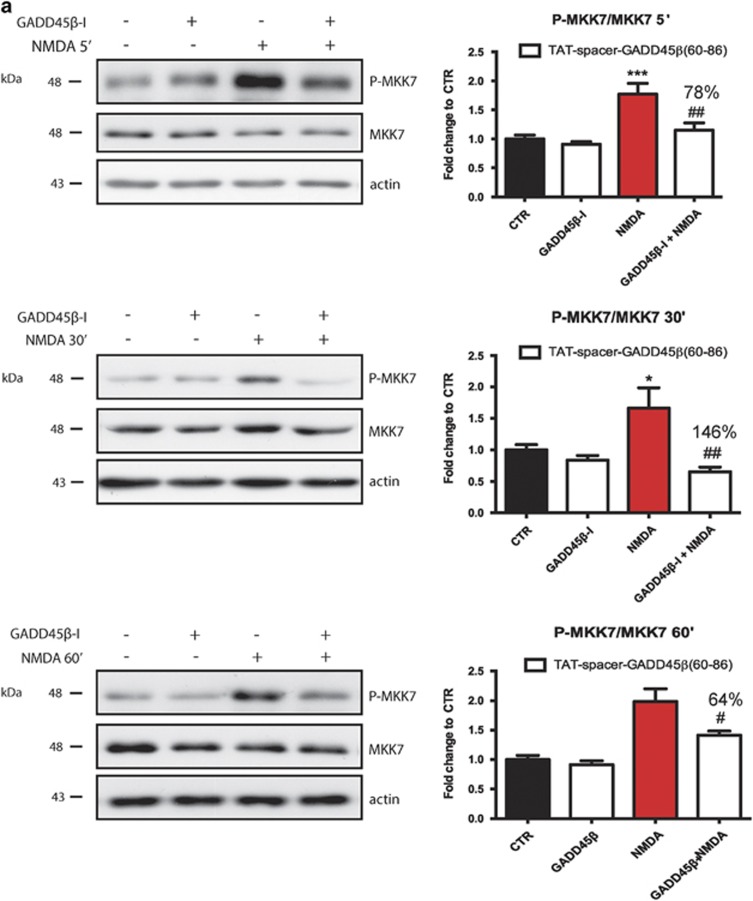

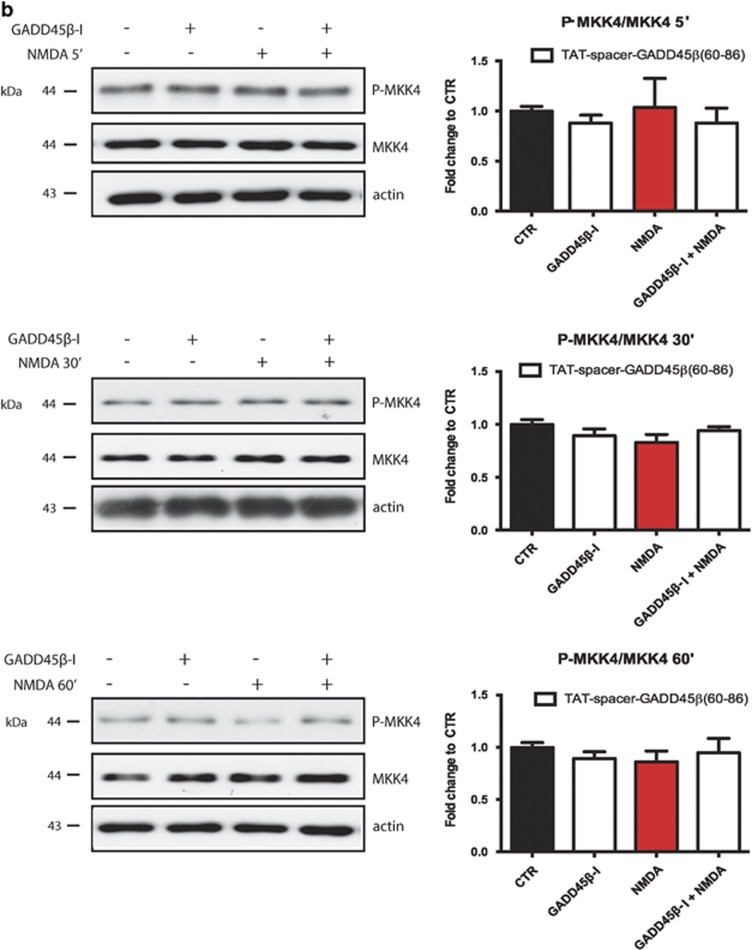
TAT-spacer-GADD45*β*_60–86_ peptide specifically prevents NMDA-induced MKK7 activation without interfering with MKK4 pathway. (**a**) Western blotting and relative quantification of P-MKK7/MKK7 ratio in 12 DIV cortical neurons treated with TAT-spacer-GADD45*β*_60–86_ (5 *μ*M), 100 *μ*M NMDA, or the combination of the treatments for 5 (upper panel), 30 (middle panel) and 60 min (lower panel). Actin was used as loading control. Data are presented as mean±S.E.M. (two-way ANOVA, Tukey's *post*-*hoc* test, *P<0.05 CTR *versus* NMDA, ***P<0.001 CTR *versus* NMDA, ^#^*P*<0.05 NMDA *versus* NMDA+MKK7I, ^##^*P*<0.001 NMDA *versus* NMDA+MKK7I, *n*=6). (**b**) Western blotting and relative quantification of P-MKK4/MKK4 ratio in 12 DIV cortical neurons treated with TAT spacer-GADD45*β*_60-86_ peptide (5 *μ*M), 100 *μ*M NMDA, or the combination of the treatments for 5 (upper panel), 30 (middle panel) and 60 min (lower panel). Actin was used as loading control. Data are presented as mean±S.E.M. (two-way ANOVA, interaction 40.05, *n*=6)

We tested the specificity of the TAT-spacer-GADD45*β*_60–86_ peptide for MKK7. Application of NMDA for 5, 30 and 60 min triggers MKK7 activation in neurons^3^ by increasing its phosphorylation by 1.7-, 1.5- and 1.9-fold, respectively, confirming its pivotal role in excitotoxicity (two-way ANOVA, Tukey's *post-hoc* test, *P<0.05 CTR *versus* NMDA and ***P<0.001 CTR *versus* NMDA) (Figure 4a). Treatment with the TAT-spacer-GADD45*β*_60–86_ peptide abolishes MKK7 phosphorylation in cortical neurons, inhibiting the kinase activity after either 5, 30 or 60 min of NMDA application by 78, 146 and 64%, respectively (two-way ANOVA, Tukey's *post-hoc* test, ##*P*<0.01 MKK7I+NMDA *versus* NMDA) (Figure 4a). On the contrary, NMDA application does not affect MKK4 activation and treatment with TAT-spacer-GADD45*β*_60–86_ peptide has no effect on MKK4 pathway (two-way ANOVA, interaction >0.05) (Figure 4b).

### MKK7 inhibition is protective in two *in vivo* models of cerebral ischemia

The TAT-spacer-GADD45*β*_60–86_ inhibitor peptide (referred as GADD45*β*−I) was tested in the MCAo and the thromboembolic ischemia model in rats.

#### MCAo model

**Figure 5:**
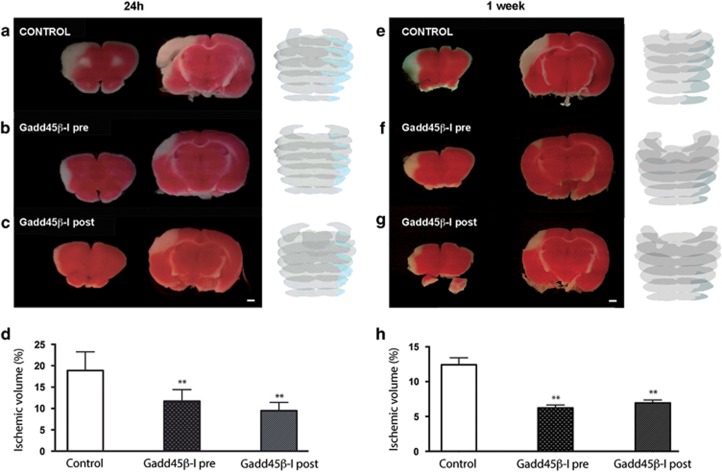
GADD45*β*-I protects against MCAO. Rats were injected with (**a**) vehicle or with (**b**) GADD45*β*-I (11 mg/kg) 30 min before lesion or with (**c**) GADD45*β*-I (11 mg/kg) 6 h after lesion. All groups were killed 24 h after lesion. Brains were extracted and colored with TTC staining to reveal ischemic areas. Ischemic volume was measured for all the three experimental groups with NIH ImageJ analysis software. Images 4A–C are TTC-stained slices at different levels of a representative animal for each different groups (scale bar 1mm). In addition, we show a full reconstruction of the lesion size (blue shade on gray) obtained with contour analysis through Neurolucida software. (**d**) Percentage of ischemic volume for the two groups (GADD45*β*-I 30 min before lesion *n*=6 and GADD45*β*-I 6 h after lesion *n*=5) of treated animals was compared with untreated animals (*n*=6). Data represent mean±S.D.; one-way ANOVA, Dunnett's *post-hoc* test, **P<0,01. Rats were injected with (**e**) vehicle 30 min before lesion or with (**f**) GADD45*β*-I (11 mg/kg) 30 min before lesion, or with (**g**) GADD45*β*-I (11 mg/kg) 6 h after lesion. All groups were killed 24 h after lesion. Brains were extracted and colored with TTC staining to reveal ischemic areas. Ischemic volume was measured for all the three experimental groups with NIH ImageJ analysis software. Images 4A–C are TTC-stained slices at different levels of a representative animal for each different groups (scale bar=1 mm). In addition, we show a full reconstruction of the lesion size (blue shade on gray) obtained with contour analysis through Neurolucida software. (**h**) Percentage of ischemic volume for the two groups (GADD45*β*-I 30 min before lesion *n*=5 and GADD45*β*-I 6 h after lesion *n*=5) of treated animals was compared with untreated animals (*n*=5). Data represent mean±S.D.; one-way ANOVA, Dunnett's *post-hoc* test, **P<0.01

We used this ischemic model to perform most of the experiments. Two different paradigms of GADD45*β*−l administration were chosen as follows: (1) 30 min before (pre-ischemic injection) and (2) 6 h after cerebral ischemia (clinical setting). Infarct area measurements were performed on 2,3,5-triphenyltetrazolium chloride (TTC)-stained brain slices 24 h after ischemia. In all animals, MCAo affected the right frontoparietal cortex areas, as well as the underlying striatum, even though moderately (Figure 5a). Pre-ischemic injection of GADD45*β*−l reduced the infarcted volume by 43% (Figures 5b–d). In fact, the percentage of ischemic volume on the whole rat brain (mean±s.d.) was 7.85±2.03% in the GADD45*β*−l-treated group (*n*=6) *versus* 13.72±4.07% in control rats (*n*=5) (**P<0.01; Figures 5b–d).

To define the therapeutic window of this inhibitor peptide, we injected GADD45*β*−l 6 and 12 h after induction of MCAo. Injection of GADD45*β*−l 6 h post ischemia reduced the infarct volume by 42%, indicating a strong protection and a great potential of GADD45*β*−l treatment (Figures 5c and d). In this case, the percentage of ischemic volume in GADD45*β*−l-treated rats injected 6 h post lesion was 8.04±1.04% (*n*=5) (**P<0.01; Figures 5c and d). On the contrary, the 12-h post-ischemic injection of GADD45*β*−l did not significantly decrease the infarct volume, indicating the failure of protection at this time point (not shown). Significant reduction in infarct size was maintained 1 week after MCAo, when pre-ischemic administration of GADD45*β*−I reduced the infarct volume by almost 50% (Figures 5e and f). The percentage of ischemic volume was 6.22±0.98% in the GADD45*β*−l-treated group (*n*=5) *versus* 12.42±2.22% in control rats (*n*=5) (**P<0.01; Figures 5f–h). Injection of GADD45*β*−l 6 h post ischemia reduced the infarct volume by 44% (Figures 5e–g). In this case, the percentage of ischemic volume in GADD45*β*−l treated rats injected 6 h post lesion was 6.95±0.93% (*n*=5) (**P<0.01; Figures 5g and h).

### MKK7-mediated neuroprotection: mechanism of action

**Figure 6:**
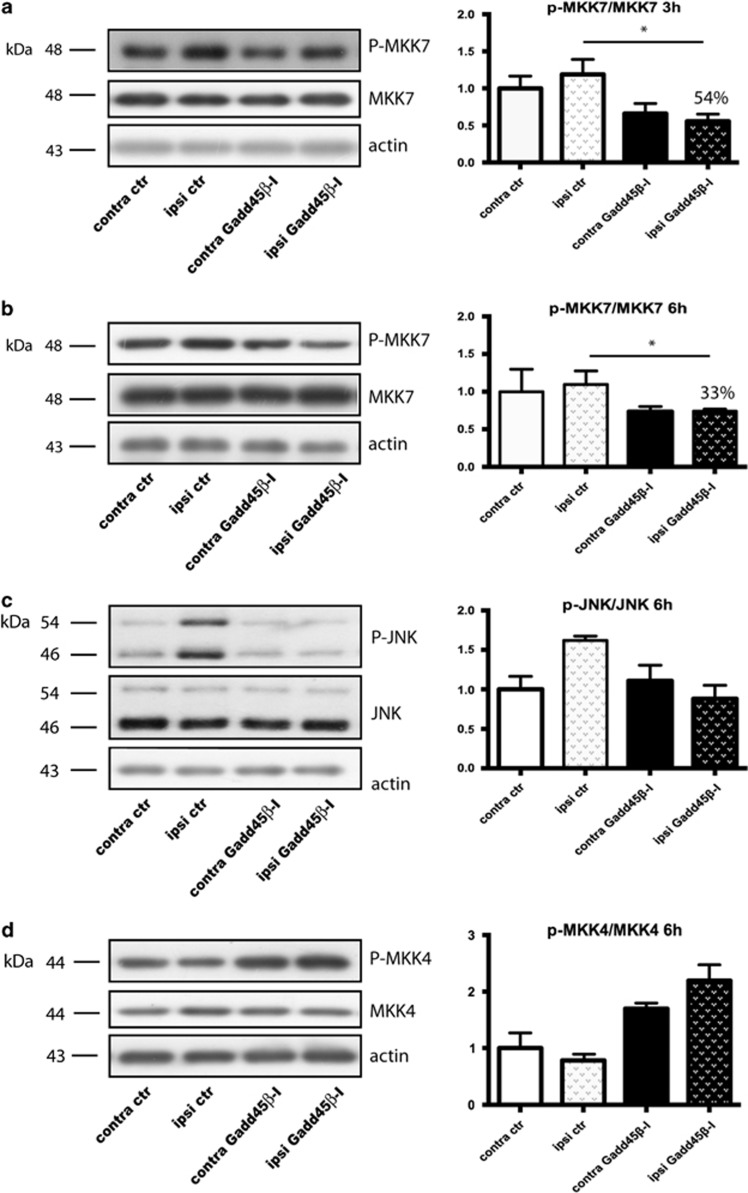
GADD45*β*-I inhibits MKK7 activation 3 h post lesion. Western blotting and relative quantification of contralateral and ipsilateral brain samples. Animals were pre-treated with GADD45*β*-I (11 mg/kg 30 min before MCAO) and killed 3 and 6 h after lesion. (**a**) Western blotting and relative quantification of P-MKK7/MKK7 ratio 3 h after lesion. Actin was used as loading control. Data are presented as mean±S.E.M. (two-way ANOVA, Tukey's *post-hoc* test *P<0.05, *n*=3) (**b**) Western blotting and relative quantification of P-MKK7/MKK7 6 h after lesion. Actin was used as loading control. Data are presented as mean±S.E.M. (two-way ANOVA, Tukey's *post-hoc* test *P<0.05, *n*=3) (**c**) Western blotting and relative quantification of P-JNK/JNK ratio 6 h after lesion. Actin was used as loading control. Data are presented as mean±S.E.M. (two-way ANOVA, interaction >0.05, *n*=3) (**d**) Western blotting and relative quantification of P-MKK4/MKK4. Actin was used as loading control. Data are presented as mean±S.E.M. (two-way ANOVA, interaction >0.05, *n*=3)

To validate the GADD45*β*−l action mechanism we characterized MKK7 activation, evaluated as P-MKK7/MKK7 ratio, in ischemic tissues from control and GADD45*β*−l-treated MCAo animals at 3 and 6 h after ischemia. As previously reported, MKK7 phosphorylation increased in the ipsilateral hemisphere after ischemia at 3 and 6 h, suggesting an early activation.^4^ Treatment with GADD45*β*−l conferred a 43% protection on infarct size, which correlated with MKK7 inhibition in treated animals compared with MCAo: in fact, rats treated with GADD45*β*−l showed a 54% reduction of P-MKK7/MKK7 ratio 3 h after ischemia (two-way ANOVA, Tukey's *post-hoc* test, *P<0.05) (Figure 6a), which persisted 6 h after ischemia (33% reduction of P-MKK7/MKK7 ratio; two-way ANOVA, Tukey's *post-hoc* test, *P<0.05) (Figure 6b). To clarify the neuroprotective mechanism of GADD45*β*−l we analyzed the levels of JNK 6 h post ischemia. At 6 h, ischemic injury leads to a pathological activation of JNK, which was reduced by application of GADD45*β*−l (Figure 6c). To verify the specificity of GADD45*β*−l for the MKK7-JNK pathway, we also analyzed the phosphorylation level of MKK4: treatment with GADD45*β*−l induces a modest, but not significant, increase in the basal activity of MKK4 (Figure 6d). These results suggest that GADD45*β*−l specifically inhibits MKK7 pathway, without blocking MKK4, and this inhibition leads to a significant neuroprotection *in vivo*, with a temporal intervention window of 6 h after cerebral ischemia.

#### Thromboembolic ischemia model

**Figure 7:**
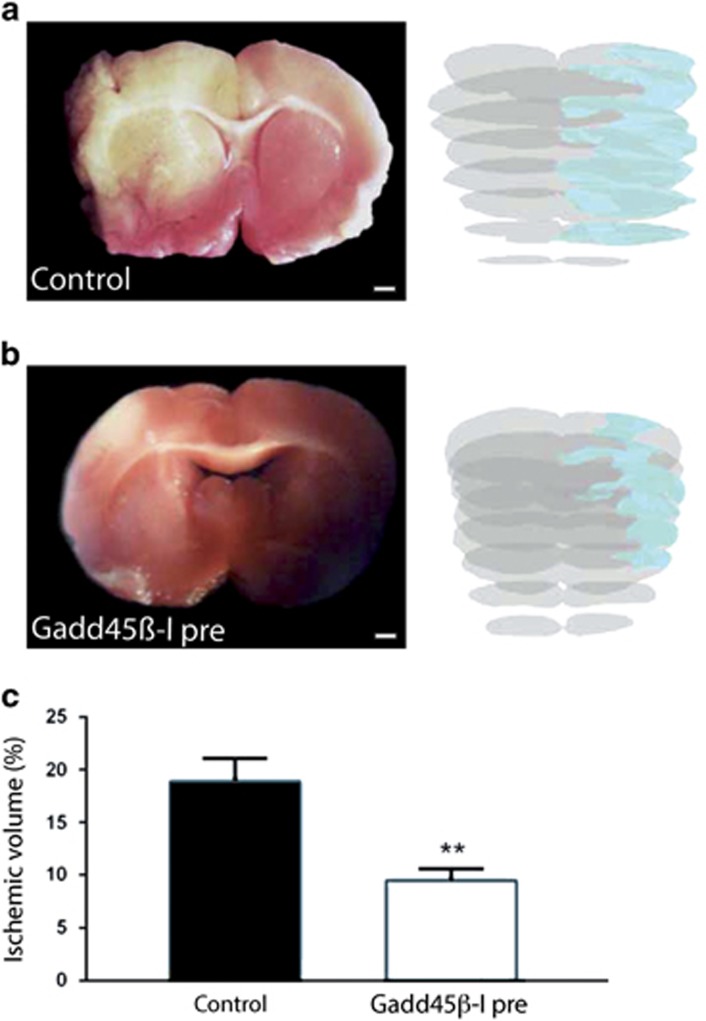
GADD45*β*-I peptide protects against thromboembolic ischemia. Rats were pre-treated with vehicle (**a**) or GADD45*β*-I (11 mg/kg, 30 min before lesion) (**b**) and killed 24 h post lesion. Brains from control (**a**) and GADD45*β*−I-treated (**b**) rats were extracted and colored with TTC staining to show ischemic region. Images 6A–B are TTC-stained representative slices of the two experimental groups (scale bar=1 mm). In addition, we show a full reconstruction of the lesion size (blue shade on gray) obtained with contour analysis through Neurolucida software. (**c**) Percentage of ischemic volume was measured with Neurolucida software and Cavalieri's geometry Treated animals were compared with untreated animals. Data represent mean±S.D. (Student's *t*-test **P<0.05, *n*=3)

To confirm the neuroprotective role of GADD45*β*−l *in vivo*, we used a second ischemic model. In this model, the GADD45*β*−I peptide was injected 30 min before the induction of ischemia. Rats treated with GADD45*β*−I showed significant improvements of the functional neuroscore 24 h after ischemia compared with the control group. Furthermore, TTC staining showed that rats treated with GADD45*β*−I peptide (*n*=3) displayed a significant decrease in the infarct volume compared with control rats at 24 h (*n*=4) (Figures 7 and b). The total mean infarct volumes were quantified using the Neurolucida software (Microbrightfield, Williston, VT, USA) and Cavalieri's geometry on TTC-stained sections 24 h after thromboembolic occlusion of the MCA (Figures 7a and b). All animals showed an ipsilateral ischemic damage, that is, in the right lateral frontoparietal cortex (Figures 7a and b). Pre-ischemic injection of GADD45*β*−l reduced the infarcted volume by 50% (Figure 7c). The percentage of ischemic lesion on the whole brain volume was 9.47±1.96% in GADD45*β*−l-treated rats *versus* 18.88±4.38% in the control group (**P<0.01) (Figure 7c). These data indicated the powerful neuroprotective effect of GADD45*β*−l against brain injury induced by thromboembolic ischemia in rats.

## Discussion

JNK is a Janus-faced molecule, which may both protect and damage neurons, regulated by the two upstream activators, MKK4 and MKK7, which are not redundant,^18, 19^ and respond differently to stress stimuli,^8, 9, 10^ representing a ‘bottleneck' in its activation.

We found that MKK7, and not MKK4, mediates the activation of JNK signaling in excitotoxic neuronal death^3^ and cerebral ischemia.^4^ Here we show, for the first time, that MKK7-specific inhibition represents a good strategy to prevent excitotoxic neuronal death and overcome possible side effects occurring in response to JNK inhibition.^13^

We designed a new MKK7 inhibitor peptide by modeling the minimal region necessary for the binding and coupling it with the TAT peptide, to allow penetration of the blood–brain barrier and neuronal membrane. A spacer was introduced between the TAT and the GADD45*β* sequences previously discovered.^9^ This allowed to functionally separate the different domains of GADD45*β*−I, guaranteeing the entry of the peptide into neurons (TAT sequence), and the ability to interact with MKK7 and inhibit its function (GADD45*β* sequence). Moreover, the spacer sequence helped in stabilizing the peptide and in increasing its solubility. The introduction of the spacer and the use of the longer sequence, which includes the seven negatively charged residues, increased the efficiency of the inhibitor peptide. In fact, *in vitro*, GADD45*β*−I (TAT-spacer-GADD45*β*_60–86_) showed a more potent neuroprotective effect against excitotoxicity, if compared with the shorter TAT-GADD45*β*_69–86_ peptide. TAT-spacer-GADD45*β*_60–86_ peptide completely protected from OGD *in vitro*, confirming the key role of MKK7 also in this model. All together, the *in vitro* results obtained with the SPR and the mutated peptide (TAT-GADD45*β*_69–86 CONTROL_) demonstrate the selectivity of GADD45*β*-I peptide for MKK7.

Finally, the peptide showed no toxicity by itself on neurons, suggesting its safety for *in vivo* treatments. To validate GADD45*β*-I for clinical applications, we tested it in two *in vivo* models of cerebral ischemia: the MCAo and the thromboembolic ischemia. Administration of the peptide before the ischemic insult prevented neuronal death, reducing the infarct volume of 43% 24 h after lesion. The neuroprotective effect was maintained also when the peptide was administered 6 h after ischemia, extending its clinical window. Currently, the utilization of tissue plasminogen activator, the only pharmacological treatment approved for ischemic stroke,^20^ is restricted by the narrow therapeutic window (3–4.5 h) and the limited efficacy, as only a sub-optimal dose (0.9 mg/kg) can be administered, owing to the risk of iatrogenic intracranial hemorrhage^21–23^, limiting treatment to an estimated 2–5% of ischemic stroke patients in the United States.^24^ Importantly, also *in vivo* GADD45*β*−I inhibited MKK7 activity and its inhibition is in agreement with the protection obtained on infarcted volumes at 24 h as well as at 1 week after lesion. Our protection *in vivo* lasts at least 1 week, thus suggesting that the effect of treatment is permanent and does not consist in a mere delay in neuronal death. The time course of phosphorylation of MKK7, that is, of its activation, is compatible with the administration time of GADD45*β*−I, that is, 3–6 h after the lesion. GADD45*β*−I revealed efficient in reducing the infarct size in both the *in vivo* models tested. Even though the thromboembolic model resembles the most adherent to human stroke, the MCAo is by and large the most commonly used for testing new drugs against excitotoxicity.

We are aware that notwithstanding the significant results obtained in preclinical studies by blocking specific molecular pathways, their clinical application remains poor^25, 26^ and no approved therapy exists for stroke other than thrombolysis. This failure in translation from bench to bedside has led to the recommendations on the stroke therapy academic industry roundtable (STAIR),^27^ even though clinical trials fulfilling the STAIR criteria for EPO or G-CSTS did not confirm preclinical experimentation as well.^28^ The reasons for this failure are multifarious, ranging from the plurality of mechanisms of cell death,^29^ the side effects of activity blockade to prevent excitotoxicity and the general conditions of the patient due to comorbidities.

A strategy would be blocking the excitotoxic cascade at the level of the activation of NMDA receptors by glutamate release inhibition, NMDAR antagonists, Na^+^- or Ca^++^ channel blockers. All of these had significant effects on neuronal death, but showed remarkable neurological and non-neurological side effects in clinical trials.^30^ Furthermore, a certain degree of NMDAR activation may be neuroprotective,^31^ eventually through the activation of the Akt pathway.^32^ An opposed effect has been shown for phosphatase and tensin homolog deleted on chromosome 10 (PTEN) pathway, which mediates NMDAR-induced death signaling. Its inhibition by a TAT-k13 peptide is neuroprotective against cerebral ischemia.^33^ No cross-links between the PTEN and the JNK pathways have been described thus far. The major neuroprotective effects both in experimental and in clinical conditions have been obtained with peptides targeting the PSD95-nNOS interaction (NR2B9c) in rodents,^34^ primates^35^ and, finally, in clinical trial.^35, 36^ In fact, the binding of PSD95 to the NMDAR and nNOS brings nNOS into close proximity with the NMDAR channel pore and with the Ca^++^ that fluxes through the NMDAR, thus activating the enzyme.^37, 38^

Recently, as an alternative strategy, we found that M5, a mutant of native urokinase plasminogen activator (prouPA), can reduce infarct size with a reduced risk of bleeding.^39^

In addition, preconditioning stimuli reduce the effects of cerebral ischemia,^40^ thus suggesting the administration of gaseous carbon monoxide or carbon monoxide donors (CORMs).^41^ Cell-based therapy has also been proposed to modulate neuroinflammation, replace dying neurons and stimulate brain repair by enhancing neurogenesis and angiogenesis, but it is far from clinical therapy.^42^

Our inhibitor may represent an important tool to specifically prevent the development of excitotoxicity in neurons, in the absence of side effects and with a very interesting therapeutic window. It might be associated to drugs acting on different pathways of death as well as to increase their efficacy. For example, blocking excitotoxicity could extend the therapeutic window for thrombolysis as well.

The *in vivo* results support its high therapeutic potential against cerebral ischemia. For the first time, specific inhibition of MKK7 was sufficient to prevent excitotoxicity *in vitro* as well as cerebral ischemia in two different *in vivo* models.^2, 4, 39^ This confirms that excitotoxicity/cerebral ischemia leads to activation of MKK7/JNK pathway, without activating MKK4/JNK. Importantly, GADD45*β*-I still prevented neuronal death after 1 week from the lesion, proving that the peptide really blocks neuronal death program after lesion and not just postpone it in time.

Compared with D-JNKI1 (specific inhibitor of all JNKs isoforms),^2^ GADD45*β*-I reduced the infarct volume by 45%, whereas D-JNKI1 reduced by 93% at the same temporal window (6 h); both peptides block neuronal death. The advantage of inhibiting MKK7 (GADD45*β*-I), instead of JNK (D-JNKI1), lies in the fact that the JNK physiological role, controlled by MKK4,^43^ is preserved with GADD45*β*-I treatment

## Conclusions

GADD45*β*−I is markedly neuroprotective against *in-vitro* excitotoxicity and OGD in cortical neurons, and more importantly against two experimental stroke models. GADD45*β*−I specifically decreases MKK7 phosphorylation/activation and reduces the infarct volume by 45–50% at 24 h post lesion, when injected 6 h post ischemia. GADD45*β*−I (injected 6 h post ischemia) reduces infarct volume by 49% at 1 week after lesion, proving that GADD45*β*−I is able to inhibit and not just postpone neuronal death after ischemia. We therefore conclude that this agent offers great promise for developing therapies for cerebral ischemia.

## Materials and Methods

### Modeling and docking design

Up to now the three-dimensional structure of GADD45*β* is not available and for this reason it has been obtained through homology modeling, using as a template the structure of the GADD45*α* (Protein Data Bank (PDB) ID: 2KGA)^44^ sharing 60% of identity with our sequence of interest.^14^ After the modeling, the structure obtained has been minimized for 1000 step using the Steepest Descent algorithm.^45^ The atomic coordinates of MKK7 have been solved and are available in the PDB (PDB ID: 2DYL), but the residues composing the three loops, 143–148, 282–296 and 307–314, are lacking. These regions have been modeled using the loops databank of the Swiss PDB viewer Version 4.1 program.^46^

In order to predict the GADD45*β*–MKK7 complex, 100 runs of docking using the HADDOCK web server,^47^ in particular suitable for the protein–protein docking, have been carried out. The results of the docking runs have been clustered based on the RMSD criterion, using a 4.5-Å cutoff. The best GADD45*β*–MKK7 complex has been chosen by considering the HADDOCK score, which is calculated according to the weighted sum of various energy terms: van der Waals, electrostatic, restraints, diffusion anisotropy, dihedral angle restraints, symmetry restraints, buried surface area, binding and desolvation energies (see the HADDOCK manual for further information^47^). Docking simulations of the GADD45*β*–MKK7 have generated 10 families, where the cluster with the best HADDOCK score contains 60% of total complexes generated after the 100 runs. The best structure of this cluster has been selected for the study of the interaction between GADD45*β* and MKK7 in order to design the peptides.

### Peptide synthesis

The synthetic peptides TAT-GADD45*β*_69–86_ (aminoacidic sequence: 5′-IALQIHFTLIQSFCCDNDGRKKRRQRRR-NH_2_-3′) and TAT-spacer-GADD45*β*_60–86_ (aminoacidic sequence: 5′-DEEEEDDIALQIHFTLIQSFCCDNDAGAGAAAGRKKRRQRRR-NH_2_-3′) were prepared by solid-phase peptide synthesis method using a multiple peptide synthesizer (SyroII, MultiSynTech GmbH, Witten, Germany) on 4-(2',4'-dimethoxyphenyl-Fmoc-aminomethyl)-phenoxyacetamido norleucyl-MBHA resin (100–200 mesh) (Novabiochem). The fluoren-9-ylmethoxycarbonyl (Fmoc) strategy^48^ was used throughout the peptide chain assembly, using *O*-(7-Azabenzotriazol-1-yl)-*N*,*N*,*N*,*N*-tetramethyluronium hexafluorophosphate as the coupling reagent.^49^ The side-chain-protected amino acid building blocks used were as follows: *N*-α-Fmoc-N*ω*-(2,2,4,6,7-pentamethyldihydrobenzofuran-5-sulfonyl)-L-arginine, *N*-α-Fmoc-γ-tert-butyl-L-glutamic acid, *N*-α-Fmoc-*β*-tert-butyl-L-aspartic acid, *N*-α-Fmoc-*O*-tert-butyl-L-serine, *N*α-Fmoc-*O*-tert-butyl-L-threonine, *N*-α-Fmoc-*N*ɛ-(tert-butyloxycarbonyl)-L-lysine, *N*-α-Fmoc-*N*(im)-trityl-L-histidine, *N*-α-Fmoc-*N*-γ-trityl-L-glutamine, *N*-α-Fmoc-*S*-trityl-cystine and *N*-α-Fmoc-*N*-*β*-trityl-L-asparagine. Cleavage of the peptides was performed by reacting the peptidyl resins with a mixture containing TFA/ethanedithiol/phenol 5% for 2.5–3 h. Crude peptides were purified by a preparative reverse-phase HPLC. Molecular masses of the peptides were confirmed by mass spectroscopy on a MALDI TOF–TOF mass spectrometer (model 4800, AB Sciex, Framingham, MA, USA). The purity of the peptides was in the range 90–95% as evaluated by analytical reverse-phase HPLC.

### SPR analyses

Binding studies were performed on a ProteOn XPR36 Protein Interaction Array system (Bio-Rad Laboratories, Hercules, CA, USA). Detection of GADD45*β*_69–86_ interaction with MKK7 was performed by immobilizing ~5000 resonance units (RUs) of human recombinant full-length MKK7 (residues M1-R419, as in NCBI/Protein entry NP_660186.1; ProQinase GmbH, Freiburg, Germany) on the surface of a sensor chip (GL-C chip, Bio-Rad) by amine-coupling chemistry. GADD45*β*_69–86_ molecules were then perfused over the chip for 150 s to allow association, followed by 500 s buffer (PBST) wash to monitor dissociation. Signals were normalized to control channels containing ~5000 RUs of bovine serum albumin or no proteins. The resulting sensorgrams were fitted to a heterogenous ligand binding model using the ProteOn analysis software, to obtain the corresponding association and dissociation rate constants (*k*_on_ and *k*_off_, respectively) and the equilibrium dissociation constant (*K*_D_).

### Cortical neuronal culture

Cortical pieces were dissected from the brains of 2-day-old rat pups, incubated with 200 U of papain for 30 min at 34 °C and, after trypsin inhibitor (Sigma Aldrich, St Louis, MO, USA) treatment (1%, 45 min at room temperature), were mechanically dissociated. Neurons were then plated at densities of ~5 × 10^5^ cells/plate on 3.5-mm tissue culture dishes (BD, San Jose, CA, USA) and 5 × 10^4^ cells/plate on 95-well culture plates (SPL Life Sciences, Pocheon, South Korea) all pre-coated with 10 *μ*g/ml poly-D-lysine (Sigma Aldrich) and 2 *μ*g/ml laminine (Life Technologies, Gaithersburg, MD, USA). The plating medium consisted of Neurobasal Medium (Life Technologies) supplemented with 2% B27 (Life Technologies), 50 mM glutamine (Life Technologies) and 1000 U/ml penicillin/streptomycin (Life Technologies). Experiments were performed after 11–13 days in culture, at which time the neurons had elaborate axonal and dendritic arbors, and had formed many synapses. GADD45*β* inhibitors were added to the dishes at the desired concentrations, 30 min before NMDA treatment (100 *μ*M, Abcam, Cambridge, UK).

All experimental procedures on animals were done in accordance with the European Communities Council Directive of 24 November 1986 (86/609/EEC) and all efforts were made to minimize animal suffering.

### Oxygen–glucose deprivation

OGD was performed on cortical neurons maintained in an Hypoxia Incubator Chamber (StemCell Technologies, Vancouver, BC, Canada) for 12 h; during the first 4 min, oxygen in the chamber was replaced by a mixture of 95% N_2_ 5% CO_2_; glucose deprivation was realized by substituting culture medium with glucose-free Neurobasal Medium (Life Technologies).

### Peptides administration

All treated rats underwent ICV administration (−1 AP, +2 ML, −3 DV), 30 min before or 6 h post lesion, of GADD45*β*-I (11 *μ*g/*μ*l) ipsilaterally to the lesion. Animals were anesthetized with isoflurane (4% during induction, then maintained with 1.5%) in a mixture of 30:70 O_2_/N_2_O and placed in a Stereotaxic Apparatus (Stoelting, 51600, Wood Dale, IL, USA). Injection (2 *μ*l volume) was made with a graduate glass pipette through an osmotic pump to minimize side effects.

### LDH cytotoxicity assay

Neuroprotection was evaluated by an LDH assay. LDH released into the bathing medium 5–24 h after NMDA was measured using the Cytotox 96 non-radioactive cytotoxicity assay kit (Promega, Fitchburg, WI, USA), according to the manufacturer's indications.

### Western blot analysis

Proteins were separated by 10% SDS-PAGE and transferred to a PVDF membrane. Incubation with primary antibodies was overnight at 4 °C using the following: 1 : 1000 anti-MKK7 (4172, Cell Signaling Technology, Beverly, MA, USA), 1 : 1000 anti-P-MKK7 (4171, Cell Signaling Technology), 1 : 1000 anti-MKK4 (07-194 Upstate, Charlottesville, VA, USA), 1 : 1000 anti-P-MKK4 (9151, Cell Signaling Technology), 1 : 1000 anti-JNK (9252S, Cell Signaling Technology) and 1 : 1000 anti-P-JNK (9252S, Cell Signaling Technology). All P-antibodies, P-MKK7, P-MKK4 and P-JNK, are specific and recognize only the phosphorylated form of these proteins (they do not recognize cortical neuronal extracts dephosphorylated with alkaline phosphatase overnight). In a single experiment, a very large number of neurons were analyzed, providing very consistent results, and the blots were all normalized with respect to actin (1 : 10 000 anti-actin, 1501 Millipore, Billerica, MA, USA). Blots were developed using horseradish peroxidase-conjugated secondary antibodies (goat anti mouse IgG-HRP and goat anti rabbit IgG-HRP, both from Santa-Cruz Biotechnology, Santa Cruz, CA, USA) and the ECL chemiluminescence system (Promega).

### Quantification

The quantification of western blottings was performed using ImageQuant TL software (Amersham Biosciences, Amersham, UK) and was based on at least three independent experiments.

### Ischemic models

#### Ethical statement

Male adult Sprague–Dawley rats (Harlan, Lesmo, Italy) weighing 270–350 g were used in this study. All animal experimental procedures were approved and carried out in accordance to the European Community Council Directive 86/609/EEC (24 November 1986), Italian Ministry of Health and University of Turin institutional guidelines on animal welfare (law 116/92 on Care and Protection of living animals undergoing experimental or other scientific procedures; authorization number 17/2010-B, 30 June 2010) and *ad-hoc* Ethical Committee of the University of Turin. Free access to food and water was maintained and all efforts were made to minimize suffering and limit the number of animals used.

*Part I. Ischemia reperfusion (n=43).* The MCA was cauterized by the method described by Renolleau *et al.*^50^ Rats were anesthetized with isoflurane (4% during induction, then maintained with 1.5%), in a mixture of 30 : 70 O_2_/N_2_O delivered with a face mask throughout the surgery duration. Briefly, under an operating microscope (Nikon, Sesto Fiorentino, Italy), a midline incision of the head was performed, the temporal muscle dissected and the temporal bone exposed; a burr hole was drilled very close to the zygomatic arch and the left MCA was identified. The MCA main branch was then electrocoagulated close to its origin at the junction with the olfactory branch. Thereafter, a median incision was made in the neck to expose the left common carotid artery (CCA), which was transiently occluded by a clip in order to reduce infarct size variability due to anastomoses in the MCA territory. After 90 min, the clip was removed. Successful occlusion was confirmed by progressive whitening of the cortex. Animals were initially killed 24 h post lesion. To further confirm the neuroprotective effect of our MKK7 inhibitor at a chronic condition, the same experimental groups were replicated but the animals were killed 7 days after lesion. In another set of experiments, control (*n*=3) and treated (*n*=3) rats were killed 6 h post surgery, to analyze the p-JNK/JNK ratio and MKK7/4 activation by western blotting.

*Part II. Thromboembolic ischemia (n=7).* Ischemia was induced by injection of autologous blood clots in suspension into the internal carotid artery (ICA), as described by Busch *et al.*^51^ In order to prepare blood clots for embolism, femoral arterial blood from a donor rat was collected into a 20-cm long PE-50 catheter and retained for 2 h at room temperature, and subsequently at 4 °C for 22 h to allow clot formation to go to completion. Clots were pushed out of the catheter with a saline-filled syringe, rinsed several times in a Petri dish containing phosphate-buffered solution (PBS, pH 7.4), in order to remove blood cells and obtain a white clot, then inspected under the microscope to select fibrin-rich fragments. These fragments were cut into 2-mm-long pieces and transferred into a solution containing 1 mg/ml albumin in PBS, to allow clot retraction. Approximately 2 h later, 20 fibrin-rich fragments were drawn up in the albumin solution in 1-m-long PE50 catheter, taking care to maintain ~3 cm distance between clots in order to keep them apart, and all embolized into MCA origin.

#### Surgical procedures

Rats were anesthetized with isoflurane (4% during induction, then maintained with 1.75%), in a mixture of 30 : 70 O_2_/N_2_O delivered with a face mask throughout the surgery duration. After a longitudinal incision of 2 cm in length in the midline of the ventral cervical skin, left CCA, ICA and external carotid artery (ECA) were carefully dissected and exposed, avoiding any damage to the adjacent vagus nerve. Inferior thyroid and occipital arteries, branching from the ECA, were visualized and cauterized; the distal portion of ECA was ligated and cut along with the terminal lingual and maxillary artery branches, and the carotid bifurcation identified. ICA was dissected cranially up to the origin of pterygopalatin branch, which was ligated using a 6/0 suture. A 5-0 silk suture was loosely tied around the origin of ECA, and then the CCA and ICA were temporarily clamped using microvascular clips. A PE-50 catheter, containing blood clot suspension, with a modified 0.3-mm outer diameter, was introduced into the ECA stump through a small puncture and advanced 1–2 mm beyond carotid bifurcation; the 5/0 suture was tightened around the catheter to prevent backflow bleeding. The clip around ICA was removed and clots injected within 30 s, whereas CCA was still occluded. At the end of injection, the catheter was withdrawn, the ECA stump was ligated and the CCA clip removed, so that blood pressure could push clots cranially. The wound was then closed; the rat was allowed to completely recover from anesthesia and returned to its cage. The surgery was complete in ~30 min.

#### Functional neuroscore

Functional outcome was tested at 2 and 24 h by observers blinded to the pharmacological regimen. A 5-point scale described by Longa *et al.*^52^ was used (grade 0: no neurological deficit; grade 1: failure to fully extend left forepaw; grade 2: contralateral circling; grade 3: contralateral falling; grade 4: absence of spontaneous movement or unconsciousness). The neuroscore was also used to further confirm that successful thrombotic occlusion had been achieved, as the existence of initial neurological deficit is a reliable predictor for successful occlusion of the MCA;^53, 54^ only animals grade 1 or higher at 2 h were included in the study. Exclusion of animals took place before assignment into the various treatment groups.

Functional neuroscore was also assessed 7 days after surgery in animals lesioned with MCAo.

#### Determination of infarct volumes

At 24 h, the animals were euthanized, brains were removed, cooled on ice and then coronally cut into 2-mm-thick sections using a tissue slicer, starting from 2 mm caudal to the frontal tip. Sections were immediately stained with 2% TTC (Sigma) at 37 °C for 10 min, then fixed in 4% phosphate-buffered formalin as previously described.^55^ Each slice was examined for subarachnoid hemorrhage. Slices were scanned with a Coolpix camera (Nikon); infarct volume and brain edema were measured using NIH ImageJ analysis software (available at http://rsb.info.nih.gov/ij/). Ischemic volumes were calculated as the sum of infarcted area (in mm^2^) multiplied by slice thickness (~2 mm). Edema volumes were calculated by subtracting the contralateral hemisphere volume from the ischemic hemisphere volume; for edema correction, the equation ischemic volume × contralateral hemisphere/ipsilateral hemisphere volume was used, as previously described.^56^ Seven 2-mm-thick slices were measured for each brain.

### Statistical analysis

Statistical analysis was done using Stat-View software. Data were calculated as mean ±S.E. Differences between groups were compared using Student's *t-*test or one-way ANOVA and two-way ANOVA, followed by Dunnett's or Tukey's test. *P*-values <0.05 were considered significant.

## Abbreviations

JNK: c-Jun-N-terminal kinase
GADD45*β*: growth arrest and DNA damage-inducible 45*β*
MKK7: mitogen-activated protein kinase kinase 7
MKK4: mitogen-activated protein kinase kinase 4
MCAo: middle cerebral artery occlusion
NMDA: *N*-methyl-D-aspartate
OGD: oxygen–glucose deprivation
SPR: surface plasmon resonance
