# Supplementary figures and images for: Exploring the role of MKK7 in excitotoxicity and cerebral ischemia: a novel pharmacological strategy against brain injury
Source: Cell Death Dis. 2015 Aug 13;6(8):e1854–. doi: 10.1038/cddis.2015.226 (PMC4558515; doi:10.1038/cddis.2015.226)

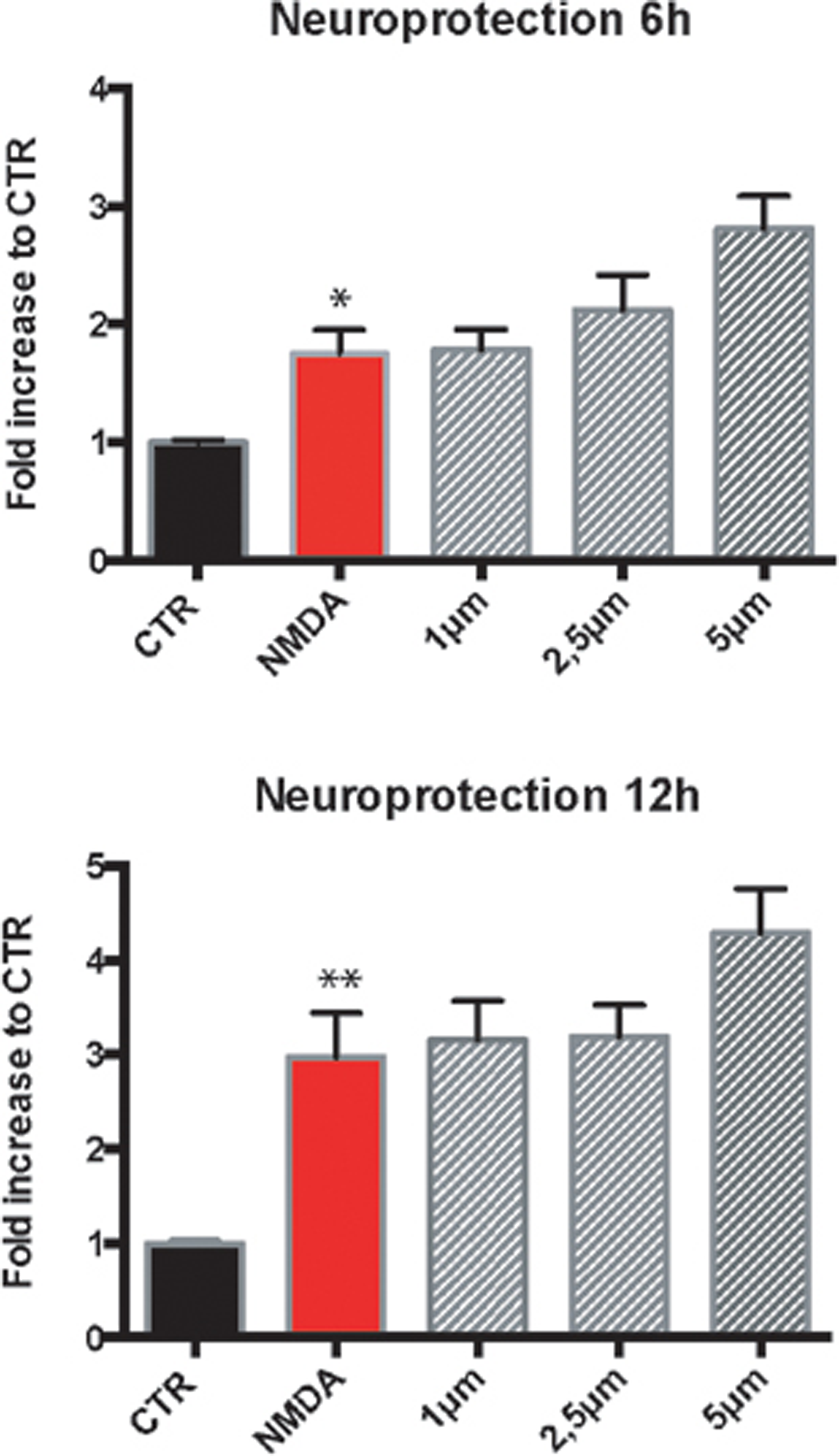

Supplement: Supplementary Figure 1 [file cddis2015226x1.tif]
