# Supplementary material for: Exploring the role of MKK7 in excitotoxicity and cerebral ischemia: a novel pharmacological strategy against brain injury
Source: Cell Death Dis. 2015 Aug 13;6(8):e1854–. doi: 10.1038/cddis.2015.226 (PMC4558515; doi:10.1038/cddis.2015.226)
Supplement: Supplementary Information [file cddis2015226x2.doc]

**Supplementary figure 1**

LDH assay performed on 12 DIV cortical neurons to evaluate the ability of TAT-spacer-GADD45β_60-86 control_ peptide (1, 2.5 and 5μM) to protect against 100μM NMDA-induced excitotoxicity *in vitro*, for 6 (upper panel) and 12 (lower panel) hours. Data are presented as mean ± S.E.M. (One-way ANOVA, Tukey post hoc test, *p<0.05 NMDA vs CTR, **p<0,01 NMDA vs CTR n=3).
